# When policy and psychology meet: Mitigating the consequences of bias in schools

**DOI:** 10.1126/sciadv.aba9479

**Published:** 2020-10-16

**Authors:** Jason A. Okonofua, Amanda D. Perez, Sean Darling-Hammond

**Affiliations:** 1 Department of Psychology, University of California, Berkeley, 2121 Berkeley Way West, Berkeley, CA 94703, USA.; 2 Goldman School of Public Policy, University of California, Berkeley, 2607 Hearst Avenue, Berkeley, CA 94720, USA.

## Abstract

Harsh exclusionary discipline predicts major negative life outcomes, including adult incarceration and unemployment. This breeds racial inequality because Black students are disproportionately at risk for this type of discipline. Can a combination of policy and psychological interventions reduce this kind of discipline and mitigate this inequality? Two preregistered experiments (*N*_experiment1_ = 246 teachers; *N*_experiment2_ = 243 teachers) used an established paradigm to systematically test integration of two and then three policy and psychological interventions to mitigate the consequences of bias (troublemaker labeling and pattern perception) on discipline (discipline severity). Results indicate that the integrated interventions can curb teachers’ troublemaker labeling and pattern prediction toward Black students who misbehave in a hypothetical paradigm. In turn, integration of the three components reduced racial inequality in teachers’ discipline decisions. This research informs scientific theory, public policy, and interventions.

## Teaser

This research explores a previously unidentified integration of approaches to mitigate racial inequity in teachers’ discipline decisions.

## INTRODUCTION

Decades of research have documented devastating and lasting effects of exclusionary discipline (e.g., suspensions that remove students from learning environments) on children and adolescents. Not only is exclusionary discipline ineffective in improving educational outcomes, but it is also associated with long-term harms that include poor future educational attainment, long-term unemployment, low lifetime earnings, and incarceration (*1*). Together, these long-term effects of suspensions are associated with a substantial economic burden to society (*2*). Ample research has also documented disparities in exclusionary discipline. This research shows that boys, Latinx children, Native American children, children with disabilities, and nonheterosexual children are at a higher risk of suspensions than their peers (*3*). Some of the largest disparities exist between Black and White children. Black students are 3.8 times more likely than White students to be suspended from school (*4*).

Recent experimental research suggests that bias can contribute to disproportionate discipline rates. In a series of experiments, researchers (*5*) asked teachers to read about two misbehaviors by a student that teachers were primed to believe was either Black or White. When the student was seemingly Black, teachers endorsed more severe disciplinary responses, particularly for the second offense, and were more likely to believe that the student would be suspended in the future. This “Two-Strikes” paradigm provides causal evidence that teachers and students face a context of heightened risk for racial bias, which shapes disciplinary outcomes (*1*).

**Fig. 1:**
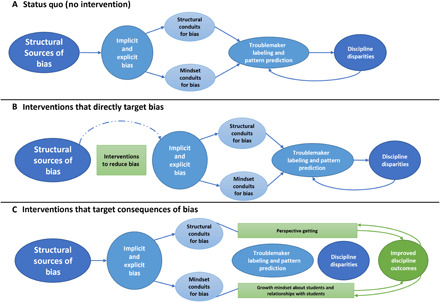
Consequences of bias on discipline decisions without or with structural and psychological approaches for lasting change. (**A**) Structural sources induce implicit and explicit bias among teachers. Structural conduits (the inability to get students’ perspectives) and mindset conduits (fixed beliefs about students and relationships) allow bias to breed troublemaker labeling and pattern prediction, leading to discipline disparities and causing a negative cycle. (**B**) Typical interventions attempt to shift discipline outcomes by mitigating bias itself. However, because structural sources of bias are overwhelmingly powerful and mechanisms by which bias acts are not affected, discipline outcomes do not shift, and the negative cycle continues. (**C**) The proposed model accepts that exposure to bias can be stable and instead intervenes to shift the structures and mindsets through which bias acts. It reduces discipline disparities, which further improves structures and mindsets, creating a virtuous cycle.

How might one mitigate the effects of racial bias on discipline? Research shows that bias itself is difficult to curb. The association between Black race and “bad” or “troublemaker” is pervasive and entrenched in the United States (*6*). For example, researchers have demonstrated that brief, nonverbal communication transmitted from television alone can increase a person’s anti-Black bias (*7*). This may explain why strategies to “debias” people typically fail (*8*). For example, in a recent study, researchers (*9*) found that none of 17 interventions consistently reduced explicit bias and most interventions (e.g., consider racial injustice) were ineffective at reducing implicit bias. These results suggest that “debiasing” may have modest, if any, effects in real-world contexts such as classrooms (see Fig. 1B). Bias can be difficult, but not impossible, to combat. It can be beneficial to strategically integrate intervention approaches across multiple fields of research. Following the debiasing intervention tournament, researchers (*10*) conducted correlational research that showed that the lack of promising effects could be due to a lack of systemic or structural changes (e.g., lack of faculty diversity in an education setting) surrounding the individuals. Might structural interventions mitigate race disparities in discipline decisions?

In response to the importance of structural characteristics in determining discipline outcomes, many schools and districts have implemented policies designed to engender productive structural shifts. Some structural shifts involve top-down mandates that shift teachers’ decisional architecture. For example, in an effort to curb reliance on, and disparities in, exclusionary discipline, many school districts have limited the use of suspensions and expulsions, particularly for willful defiance. Other structural shifts stem from policies that provide teachers with tools and time necessary to individuate students, understand their perspectives, and improve student-teacher relationships. In 2014 alone, the U.S. Department of Education provided $40 million in funding to a dozen states and more than 70 school districts to implement policies that could improve school climates. Via these and other funding streams, states, districts, and schools have implemented a range of structure shifting interventions such as restorative practices (RPs), positive behavioral interventions and supports, and professional development to improve cultural competency (CC). To be sure, in many instances, these structural interventions include trainings that could ostensibly shifts teachers’ mindsets. In many RP trainings, for example, teachers are encouraged to adopt the belief that instances of student misbehavior stem largely from social distance and that the most efficient curative measure for such misbehavior is to address social harm and distance. However, in many cases, schools and districts implement RP as a diversion program, taking a subset of students who would have been suspended under prior discipline regimes and instead guiding them through a restorative process that encourages them to repair harm done to their peers. These diversion programs may not involve mindset shifts for teachers. Fundamentally, then, while structure shifting interventions may at times lead to mindset shifts, they are often not designed specifically to achieve them. Moreover, as we will discuss more below, typical structure shifting interventions are not designed to achieve the kinds of mindset shifts that, research suggests, can reduce discipline gaps (*5*). Expectedly, then, while research suggests that these structure shifting approaches can yield improvements in student-teacher relationships and reduce the frequency of severe discipline, these approaches have yielded mixed results in terms of their ability to reduce disparities in discipline based on student race (*11*).

Together, this research suggests that both psychological (e.g., mindset) and structural (e.g., policy) interventions, when implemented in isolation, are at best inconsistent and at worst ineffective tools for achieving equity in school discipline. Moreover, it suggests that combining psychological and structural strategies may yield superior outcomes. However, research has yet to provide a systemic investigation of how structural and psychological interventions could effectively be integrated.

Recent research has shown that regional anti-Black bias is associated with racial inequality in exclusionary discipline throughout the United States (*12*). While there is a link between bias and discipline disparities, approaches that seek to directly reduce racial bias lack sustained real-world benefits and thus are poor candidates for combatting discipline disparities. We propose that focus should be placed on mitigating the negative consequences of racial bias as opposed to mitigating racial bias itself. Phrased another way, racial bias causes harm through processes that, if interrupted, may sap bias of much of its negative potential. The present research investigates whether interventions that target the processes by which bias affects perception and decision-making can alleviate inequalities in discipline outcomes.

By what process might bias affect discipline? The Two-Strikes research pinpointed a process by which race effects on discipline emerge. Teachers were more likely to view Black students as troublemakers (troublemaker labeling) and to see Black students as engaging in a pattern of misbehavior (pattern prediction), and for any student deemed a troublemaker, teachers wanted to respond with harsher discipline (*5*). Interventions that target this process might, therefore, more effectively mitigate disparities in discipline decisions.

On the basis of the theoretical considerations described above, interventions are expected to be more effective when they target consequences of bias and strategically integrate both structural and mindset approaches. The present research tests whether a combination of structural (policy) and psychological (mindset) intervention strategies can disrupt the process by which anti-Black bias contributes to racial disparities in discipline. These strategies are (i) getting perspective from students, (ii) believing students’ behavior can improve, and (iii) believing relationships can improve, which combine policy (i) and mindset (ii and iii) approaches (see Fig. 1C).

### Policy: Getting perspective

Classic research has shown that the likelihood for bias to affect one’s perception or decision-making is significantly reduced when one has more individuating information (*13*). This suggests that consequences of bias may be mitigated when a teacher has the opportunity to learn more about a student and get context for the student’s behavior (*1*). School policies (e.g., RP) and skill building (e.g., CC) that prioritize opportunities and capacities for a teacher and a student to discuss their relationship and behaviors can reduce the likelihood of discipline problems in the future (*11*). Together, might learning more about a misbehaving Black student’s perspective contribute to a reduction in the likelihood that a teacher will view the student as a troublemaker (experiment 1) or view the misbehavior as a pattern (experiment 2)? In turn, might it help mitigate racial inequality in teachers’ discipline decisions (experiment 2)?

### Mindsets: Incremental theories

Research suggests that the belief in the possibility of growth, a “growth mindset,” can improve an individual’s responses to conflict. This can be relevant to combating the troublemaker labeling and pattern prediction processes in two ways: (i) reduce the likelihood that a teacher views a misbehaving student’s personality as incapable of change and (ii) reduce the likelihood that a teacher views themselves as unable to change their relationships with their students. In prior research, encouraging participants to believe that personalities can change reduced the likelihood of aggressive retaliation and increased prosocial behavior toward a person believed to be disrespectful (*14*). This research is particularly relevant in the context of discipline disparities, as teachers suspend Black students at a disproportionate rate for “disrespect” (also termed “willful defiance”) (*3*). Might teachers be less likely to label a misbehaving Black student as a troublemaker if they are encouraged to believe that students’ behaviors can and do improve (experiments 1 and 2)? In addition, research has shown that it is possible to shift a person’s faith in their ability. The shift to a belief that ability can develop (growth mindset) leads to resiliency and effort to improve that ability (*15*). When teachers develop or remember a growth mindset about their ability to improve relationships with students, might they become more resilient to conflict and commit more effort to sustaining high-quality relationships with students they would otherwise view as troublemakers (e.g., misbehaving Black students) (experiment 2)?

In the present research, we tested whether a combination of getting perspective and exposure to relevant incremental theories can mitigate the consequences of bias on discipline decisions. We call this combination of approaches a “bias-consequence alleviation” (BCA) intervention. The present research sought to determine how the following components can be integrated to reduce the process by which bias contributes to racial inequality in discipline decisions: (i) getting a misbehaving student’s perspective, “student perspective”; (ii) belief that others’ personalities can change, “student growth”; and (iii) belief that one’s own ability to sustain positive relationships can change, “relationship growth.” Can a combination of these three components curb troublemaker labeling and pattern prediction responses to a Black student’s misbehavior (experiments 1 and 2) and, in turn, mitigate disproportionate discipline (experiment 2)?

For consistency across experiments and connection to real-world outcomes (i.e., suspension rates), we used the Two-Strikes paradigm in each experiment (*5*). Participants were prompted to imagine two misbehaviors by a hypothetical Black student (named Darnell or DeShawn). First, teachers read and responded to the following randomly counterbalanced scenario:

Darnell comes in late to your class during test day. You ask for his tardy pass. He does not respond. You ask again for him to give you his tardy pass. He slams it on your desk. Then, while the class is taking the test, Darnell makes a lot of noise stomping to his desk.

Next, teachers were told that the student misbehaved three days later and read and responded to the following scenario:

Today, Darnell is upset because you “bother” him when he “wants to sit quietly and do nothing”. And he says that you should just leave him alone. So you give him reading assignments and just busy work. But Darnell calls you “crazy” and doesn’t do anything you give him.

Experiment 1 was an initial test of whether, together, a structural intervention (student perspective) and a mindset intervention (student growth) could effectively be integrated to curb consequences of bias (e.g., troublemaker labeling). Experiment 2 tested whether combining an additional mindset intervention (relationship growth) with the first two interventions could mitigate both consequences of bias (troublemaker labeling and pattern prediction). Experiment 2 manipulated the student’s race to be Black or White (named Greg) to test whether the BCA intervention effects mitigate previously evidenced differences in how teachers respond to misbehavior dependent on the student’s race. See Table 1 for a sequential description of the procedure for experiment 2. Hypotheses that the BCA intervention will improve responses to the student after his second misbehavior were preregistered. This means that these expectations were publicly made clear before the reported experiments were run, and thus, analyses based on these hypotheses are rigorous and appropriate.

**Table 1:** Sequential description of procedures for experiment 2.

	**Treatment**	**Control**
Intervention 1	Read article and answer questions that encourage endorsement of student growth	Read article and answer questions that encourage endorsement of student technology use
Intervention 2	Read article and answer questions that encourage endorsement of relationship growth	Read article and answer questions about relationships being fixed
Prime 1	Read about a misbehavior incident involving either a White (Greg) or Black (Darnell) student (2b) or simply involving a Black student (2a)	
Data collection 1	Answer questions regarding how troubled they feel about the student’s behavior, how severely they would discipline the student, etc.	
Intervention 3	Imagine and answer questions about getting student’s perspective (talking to him and finding out he has worried about belonging at school)	Imagine and answer questions about writing in a journal
Prime 2	After being told to imagine 3 days have passed, read about a second misbehavior incident involving the same student as depicted in prime 1	
Data collection 2	Answer questions regarding how troubled they feel about the student’s behavior, how severely they would discipline the student, etc.	

After each misbehavior, teachers were asked how troubled they felt and how severely they thought the student should be disciplined. They were also asked their likelihood to say that the student was a troublemaker (troublemaker labeling), extent to which the misbehavior was indicative of a pattern (pattern prediction), and the perceived strength of their relationship with the student. Last, to gauge downstream consequences of the process, teachers were asked the extent to which they would expect the student to get suspended in the future. Two additional questions were included for exploratory purposes (see the Supplementary Materials). See Table 2 for the list of variables included in each experiment. Scales for all outcomes range from 1 (“not at all”) to 5 (“extremely”). Before any experiment began, approval from the Institutional Review Board at the University of California at Berkeley was given.

**Table 2:** List of independent and dependent variables across experiments.

		**Experiment**		
		**1**	**2a**	**2b**
**Interventions received by**				
**Treatment group**	**Control group**			
Student perspective	Journal writing	✓	✓	✓
Student growth	Technology-engagement	✓	✓	✓
Relationship growth	Relationship-fixed		✓	✓
**Student races studied**				
Black (i.e., named Darnell or DeShawn)		✓	✓	✓
White (i.e., named Greg)				✓
**Hypothesized outcomes**				
Feeling troubled by student’s conduct↓		✓	✓	✓
Discipline severity↓		✓	✓	✓
Troublemaker labeling↓		✓	✓	✓
Pattern prediction↓		✓	✓	✓
Strength of relationship with student↑		✓	✓	✓
Predicting student will be suspended in the future↓			✓	✓
Feeling personal responsibility for student’s conduct↑			✓	✓

## RESULTS

**Fig. 2:**
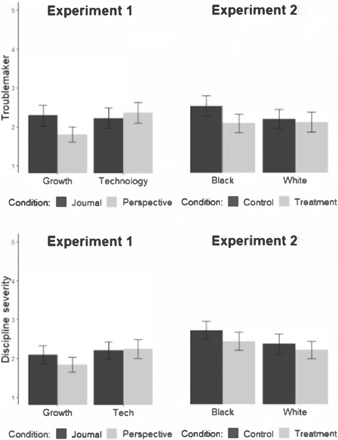
Experiments 1 and 2 effects on troublemaker labeling and discipline severity. Labels are as follows: experiment 1 (Growth, student growth treatment; Tech, technology control; Journal, journaling control; Perspective, student perspective treatment) and experiment 2 (Control, journaling control, technology control, and relationship-fixed control; Treatment, student perspective, student growth, and relationship growth; Black, student named Darnell; White, student named Greg). The error bars signify 95% CIs.

In experiment 1, 246 K-12 teachers were randomly assigned to a 2 (student growth treatment versus technology control) × 2 (perspective treatment versus journaling control) factorial design. As predicted in preregistration (https://osf.io/kysmv/?view_only=01e64c76c62245ac825351107db200ce), a linear regression revealed a significant interaction of the interventions (student perspective and student growth) such that when combined, they led teachers, after the second misbehavior, to feel less troubled [*b* = −0.11, *se* = 0.05, *t*(3, 242) = −2.20, *P* = 0.03, 95% confidence interval (CI) (−0.21 to −0.02)] and be less likely to label the Black student as a troublemaker [*b* = −0.16, *se* = 0.06, *t*(3, 241) = −2.46, *P* = 0.01, 95% CI (−0.28 to −0.03)], as compared to any other condition. Furthermore, main effects emerged for the student growth treatment such that it led teachers to more likely feel able to build a strong relationship with the student [*b* = 0.16, *se* = 0.06, *t*(3, 242) = 2.49, *P* = 0.01, 95% CI (0.3 to 0.28)] and to want less severe discipline [*b* = −0.13, *se* = 0.06, *t*(3, 242) = −2.28, *P* = 0.02, 95% CI (−0.24 to −0.12)]. However, there was no significant effect on pattern prediction (*P* = 0.26). See Fig. 2 for graphs of primary dependent variables: discipline severity and troublemaker labeling. Would these effects be enhanced by layering in the relationship growth intervention discussed above? In addition, how might these interventions shift teachers’ responses to White students?

A pilot experiment with 257 Amazon Mechanical Turk workers (experiment 2a) showed that integration of the third treatment (relationship growth) improved responses to a Black student’s misbehavior (see the Supplementary Materials). Amazon Mechanical Turk is a crowdsourcing website for businesses to hire remotely located “crowdworkers” to perform discrete on-demand tasks. It is operated under Amazon Web Services and is owned by Amazon. In the science community, it has become a pool for participants in online surveys or studies.

In experiment 2b, we tested whether the benefits of all three integrated components can be replicated with 243 teachers (as many as could be recruited before the conclusion of the academic year; mean age (*M*_age_) = 40.62 and SD = 11.16.). We also sought to confirm that the effects do not harm responses to or relationships with misbehaving non-Black (i.e., White) students. Teachers were randomly assigned to receive the integration of treatments or to receive similar activities with control themes and to read about misbehavior by a Black or White student in a 2 (procedure: treatment versus control) × 2 (student: Black versus White) between-subjects factorial design. As predicted in preregistration (http://aspredicted.org/blind.php?x=5jf2qp), independent-samples *t* tests revealed that teachers under the treatment condition, as compared to teachers under the active control condition, were less likely to label the student as a troublemaker [*t*(108.61) = −2.50, *P* = 0.01, *d* = −0.47, 95% CI (−0.81 to −0.09)], were less likely to think that the misbehavior was indicative of a pattern [*t*(112.65) = −1.88, *P* = 0.06, *d* = −0.35, 95% CI [−0.71 to 0.02)], were more likely to feel able to build a strong relationship with the student [*t*(112.45) = 3.33, *P* = 0.001, *d* = 0.62, 95% CI (0.22 to 0.85)], and were less likely to expect the student to get suspended in the future [*t*(105.07) = −2.75, *P* = 0.007, *d* = −0.51, 95% CI (−0.92 to −0.15)]. See Fig. 2 and table S3 for details about each finding. On average, teachers under the experimental condition felt somewhat less troubled by the Black student’s misbehavior [*t*(114.45) = −1.3495, *P* = 0.18, *d* = −0.25, 95% CI (−0.56 to 0.11)] and somewhat less desirous of severe discipline for the Black student [*t*(114.98) = −1.74, *P* = 0.09, *d* = −0.32, 95% CI (−0.05 to 0.69)], although neither effect was statistically significant.

Future research should investigate means to effectively improve outcomes on these two measures or why they are not relevant to benefits on key outcomes (e.g., troublemaker labeling and future suspensions). In addition, as predicted in preregistration, the treatment did not harm teachers’ responses to misbehavior by a White student. Rather, it made responses to both Black and White students more positive and productive.

## DISCUSSION

Inspired by past research in independent fields of study, we built on theory to mitigate pervasive racial inequity in societal outcomes. Namely, we tested whether (i) targeting the consequences of bias (troublemaker labeling and pattern prediction) might curb racial inequality and (ii) an integration of treatments would be most productive at strategic alleviation of those consequences of bias. Specific to the K-12 schooling context, we established the potential for an integration of policy/skill building and psychological theories to combat the effects of anti-Black bias on discipline decisions.

We find that a cohesive integration of key treatments, getting perspective and acting from a belief that student behavior and teacher-student relationships can improve, can thwart the process by which anti-Black bias contributes to racial disparities in discipline decisions. The findings show how targeting the process of bias, as opposed to bias itself, may serve as an effective way to mitigate societal disparities. This advanced theory simultaneously elevates targeted “wise” interventions (*16*) and galvanizes strategic integrations with relevant structural policies. Getting perspective from a misbehaving student takes time. It also requires that teachers have the opportunity to build deeper relationships with students. At present, many schools do not have policies or practices that empower teachers to get a misbehaving student’s perspective or to foster a deeper relationship with a misbehaving student. There are notable and instructive exceptions. For example, many schools currently utilize RPs, such as community building circles, designed to foster and deepen student-teacher relationships. Research on these practices suggests that they may improve teacher-student relationships by helping teachers proactively nurture caring relationships with students and reconcile with students following student acts of misbehavior (*17*–*21*). This research also demonstrates that professional development approaches with teachers can help them gather and master productive strategies for engaging with and getting perspectives from students, particularly Black students who have misbehaved (*18*, *21*). Last, research suggests that increasing racial diversity in the teaching force can serve as an additional structural approach to enhancing the capacity for understanding students’ perspectives and, in turn, responding to misbehavior in a less punitive manner (*22*, *23*). Greater than the sum of their parts, these hybrid approaches, structural and psychological, may lend themselves to potent, unexplored means to address societal issues.

## MATERIALS AND METHODS

### Experimental design

The sample sizes for each experiment were based on the number of teachers in the school district and power needed for meaningful analyses. In our preregistration, we did not make a rule for stopping data collection. Data collection ceased when teachers in the school district stopped volunteering to participate in each experiment. Teachers had until the end of the academic year to do so before the online portal to the survey was closed. Data exclusions followed the protocols depicted in our preregistration document, and were as follows: (i) missing data on responses to first scenario in the Two-Strikes paradigm and (ii) if teachers are able to state that the survey has to do with testing racial bias in a suspicion check question. There were no exclusions based on potential data outliers. There were no end points to select beyond those of any scale. The basic experiment (Two-Strikes paradigm) was replicated in the pilot experiment and both experiments described here. In all three experiments, effects on discipline decisions were consistent and in predicted directions.

The objective of this research was to test how the components of the BCA intervention [(i) a growth mindset about students’ potential to grow, develop, and learn better behavior; (ii) perspective taking as a strategy to improve teacher-student relationships and protect their integrity in times of conflict, misbehavior, and discipline; and (iii) a growth mindset about how relationships with students can develop and improve with effort] can mitigate the documented effects of racial bias on teachers’ responses to student misbehavior. All hypotheses were preregistered and based on past research that has found that these components, when integrated, could mitigate the consequences of bias on decision-making in the schooling context. In this research, it was important to understand responses by teachers who have real-world experience with interacting with students in K-12 education. Thus, in both experiments described in this research, only K-12 teachers were recruited to participate in the experiment. We chose to place focus on teachers with real-world experience because we suspected that it would contribute to the generalizability of the findings to real-world outcomes in schools.

A 2 × 2 factorial design was used as the experimental design for each experiment here. This provides for a rigorous test of the hypotheses based on the highest standards of science (e.g., random assignment to conditions). In each experiment, teachers were randomly assigned to each condition specified for that experiment. Participants were blinded to the hypotheses or the conditions for each experiment. The experimenters were blinded to the actual identities, beyond keys for merging datasets, of teachers assigned to conditions but were aware of the hypotheses and experimental design described in this section.

Next, we will detail the methods and materials for each experiment reported here. Each experiment followed the same procedure as the Two-Strikes paradigm (*5*). In this paradigm, participants read about two misbehaviors, committed 3 days apart, by a target student and are asked a series of questions after each misbehavior. However, different strategies were used before or during the procedure, based on the treatments tested in each experiment (see Tables 1 and 2).

Each treatment had materials for a corresponding control condition that involved a similar activity (e.g., answering questions about an article). The control condition used to correspond with the student perspective treatment was an activity in which teachers took time to write in a journal. Journal or diary writing interventions have been found to lead participants to have a more positive outlook on life [(*24*) but also see (*25*)], and the practice is not likely an uncommon practice for teachers. The corresponding control condition for the student growth treatment was a similar activity in which teachers read about how technology is useful to keep students engaged. This is the same control condition materials used to test the efficacy of the empathic mindset intervention (*1*). These control materials resulted in suspension rates similar to average rates of suspensions reported by the U.S. Department of Education. Last, the control materials used to correspond with the relationship growth treatment were a similar activity that asked teachers to read an article about how relationship quality is typically stable and to respond to questions about how that has been true in their experience. This kind of “fixed-mindset” control condition is used to directly gauge effects opposite of incremental theories.

Because the student perspective treatment occurs after the first misbehavior (see Table 1 for design of experiment 2) and effects typically only emerge after the second misbehavior in this paradigm (*5*), our analyses focus on teacher responses after the second misbehavior. For analyses of repeated measures (i.e., feeling troubled and discipline severity), we control for teachers’ responses to the initial misbehavior. Across all experiments, effects on primary outcomes (discipline severity and troublemaker labeling) remained consistent with or without controlling for teachers’ responses to the initial misbehavior.

All analyses were conducted in the statistical software R using linear regression. Each level of the intervention was effect coded −1 for the control and 1 for the treatment, respectively.

### Experiment 1

#### Participant information

Because this was a teacher sample, no exclusions were made on the basis of feedback/prompts. Two teachers did not complete all prompts. However, effects remained consistent whether they were excluded. Therefore, to establish as much power as possible in the statistical analyses, reported results do not exclude these participants. Preregistered hypotheses specified predicted effects for the second misbehavior. In past research, a “black-escalation” effect has been found such that teachers’ responses to misbehavior escalate more sharply for Black students, as compared to White students, from the first misbehavior to the second misbehavior [see (*5*)]. In the current research, the teachers only read about Black students. Thus, potential for the black-escalation effect was present across conditions. Furthermore, one condition, procedure, was applied between the first and second misbehaviors and was thus unable to produce effects on the first misbehavior. Teachers (218 female, 21 male, and 7 declined to state) were randomly assigned to conditions in a 2 (structure: student perspective versus journaling control) × 2 (mindset: student growth versus technology control) between-subjects design. The racial breakdown of our sample was as follows: 212 White, 9 Black, 8 Latinx, 8 Other, 2 Asian, and 7 declined to answer. Our sample had a *M*_age_ = 43.24 and SD = 10.35 and average years of teaching experience of *M* = 14.70 and SD = 9.20.

#### Procedure

##### Perspective

Teachers were randomly assigned to either get information about the target student’s perspective or get information about a personal journal entry and then answer questions about the experience. This manipulation occurred between teachers’ review of the first and second misbehaviors. Half of the teachers read about learning more information about the student from the student. This made the treatment about the process of getting perspective as opposed to imagining perspective (*26*). Research suggests that simply trying to take another person’s perspective may not help people understand other people better. However, people can achieve greater psychological understanding through conversation and listening. “Perspective-getting” leads to increased empathy for another person, increased sense of similarity and connection to others, better cooperation, and strengthened social bonds [see (*27*)]. This work suggests that teacher-student relationships can benefit from perspective-getting (e.g., teachers learning a Black student’s perspective) and do so in ways that can also combat conditions under which bias affects social cognition [e.g., more individuation and less ambiguity in decision-making; see (*1*)]. Between the misbehaviors, these teachers read the following:

When you get the time, you try to talk with students during off periods. A few days later, you spoke with Darnell from your class. He told you about how he likes music and plans to learn how to play multiple instruments. Darnell also talked about how he struggled with things he experienced outside of school. Sometimes he wondered if anyone even cared. And sometimes it made him feel like school wasn’t for him. He likes music and plans to learn how to play multiple instruments.

Teachers were then given the opportunity to describe what else they would do with their off period and what they would talk to the student about if they spent more time talking to him. The other half of the teachers read about their experience with writing in a journal during their off period. Between the misbehaviors, these teachers read the following:

When you get the time, you try to write in your journal during off periods. A few days later, you took time to write. You write about how you like music and some of the instruments you wish you could play. You think playing the trumpet would be fun but also difficult to learn. Sometimes you go to the music store to play around with some of the instruments, but you have never actually bought anything before.

These teachers were then given the opportunity to describe what else they would do with their off period and what else they would write in their journal if they spent more time writing in it during their off period.

##### Student growth

Teachers were also randomly assigned to either read about how misbehaving students’ personalities can improve or about how technology use can enhance student engagement. This manipulation occurred directly before teachers read about the first misbehavior. Half of the teachers read about how students and their behavior can and do change. This message drew from past research on an incremental theory of personality (*14*). These teachers read the following message taken from the treatment condition in the empathic mindset intervention (*1*):

Almost everyone has a personal story about a great teacher who influenced his or her life. For some, it’s a teacher who reached out and helped them feel both comfortable and respected in school. For others, it’s a teacher who helped them see that they could reach a higher standard, even when they doubted themselves. As teachers, these stories warm our hearts. They inspire us to create a positive setting that brings out the best in our students.

Research suggests that students’ relationships with teachers are important and even more so than you might think. Children who experience caring relationships with adults grow up to be more respectful and caring people. At home, a kind and responsive parent shows a child that their family is good and trustworthy. In school, a teacher who makes his or her students feel heard, valued, and respected shows them that school is fair and they can grow and succeed there.

Of course, creating positive relationships is not always easy, especially with middle school students. The social and biological changes of adolescence can make middle school students insecure and sensitive. However, students’ attitudes about school and behavior can and do improve when teachers successfully convey the caring and respect students crave.

Teachers were then asked to list two ways that students can become better behaved and more respectful when they have a caring and supportive relationship with a teacher. This is an adaptation of the “Saying Is Believing” technique that can solidify the delivery of intervention messages by allowing teachers to assume the role of experts as opposed to recipients of an intervention [see (*16*)].

The other half of teachers read about how technology use can enhance student learning and engagement. These teachers read the following message taken from the control condition in the empathic mindset intervention (*1*):

There are many ways of learning that come together to make a whole. Reading lessons and texts, viewing pictures and graphs, and listening to lectures are all examples of ways students can learn new information. All together, these forms of learning provide useful means for students to grow and develop in school. As teachers, it is effective to incorporate approaches that appeal to many learning styles when planning lessons, and that’s where technology can help.

Researchers have started to systematically explore the benefits of technology in effectively implementing lesson plans. The research suggests that using certain devices is more important in class than most people think. It allows teachers to help students grow by adapting to their various learning styles. It is particularly useful when presenting lectures, keeping a calendar, and managing assignments. Research finds that little additions of computer-based programs can help adapt lessons for emerging student learning styles. By better understanding technology teachers can nurture students’ growth into more organized, more motivated young adults.

Similar to the treatment condition, teachers were then asked to list two ways that students can benefit from more technology use in the classroom.

#### Measures

All questions were asked on a scale of 1 (not at all) to 5 (extremely). Following each misbehavior, teachers were asked the following questions: (i) How severe was Darnell’s behavior? (ii) To what extent is Darnell hindering you from maintaining order in the class? (iii) How irritating is Darnell? (iv) How severely should Darnell be disciplined?

Similar to previous research, the responses to the first three questions were aggregated into a measure called “feeling troubled” (*5*). After the two misbehaviors, teachers were also asked the following questions: (i) How likely is it that you would say that Darnell is a troublemaker? (ii) To what extent do you think Darnell’s behavior is indicative of a pattern? (iii) How likely is it that you will be able to build a strong relationship with Darnell? (iv) To what extent do you think Darnell is a danger to other students?

### Pilot study

#### Participant information

Since participants in this study were Amazon Mechanical Turk participants with unconfirmed identities, we removed two participants who failed attention checks, leaving a final sample size of *n* = 257 (132 females and 125 males; *M*_age_ = 34.33 and SD = 10.24). The racial breakdown of this sample was as follows: 197 White, 21 Black, 19 Asian, 17 Latinx, and 3 declined to answer. Similar to experiment 1, preregistered hypotheses only delineated predicted relationships after the second misbehavior. Experiment 2a followed the same procedures as experiment 1 (student perspective and student growth versus journaling control and technology control) with the addition of a manipulation for an incremental theory of teachers’ ability to improve relationships with students (see Table 1). Participants were randomly assigned to either get all three treatments (student perspective, student growth, and relationship growth) or all three controls (journaling control, technology control, and relationship-fixed control) in a two-cell experimental design.

#### Procedure

Participants were asked to imagine themselves as teachers at a hypothetical school. In addition to the treatments and controls from experiment 1, they were randomly assigned to either engage with information about how teachers can develop the ability to improve relationships with students, especially when they misbehave, or to engage with information about how the quality of relationships is typically stable. This manipulation occurred after the student growth manipulation and before participants read about the first misbehavior. Half of the participants read about how teachers can and do improve their relationships with students. This message drew from past research on an incremental theory of intelligence (*15*). These teachers read the following message taken from the treatment condition in the empathic mindset intervention (*1*):

Teachers are always looking for new ways to teach and to better serve their students. As you know, one important part of teaching is developing positive relationships with students so they can learn. Our own research team has been studying the role of teacher-student relationships in students’ motivation, learning, and behavior.

Through interviews and focus groups, teachers have told us that this can be difficult at times. Many times, teachers try new strategies, and students are not responsive to their efforts. This can be discouraging. However, they agree that it is important to keep trying. Try to be patient and try to try more strategies. It is a part of the process to gain students’ trust. Teachers say that over time, sometimes after many efforts, they become better at building caring relationships with students and earning their trust. In turn, students feel more motivated to behave well at school and to listen to teachers’ guidance.

Participants were then asked to describe a situation when a teacher reached out to a misbehaving student and it helped the student feel respected and motivated to behave better. They were also asked to explain why it is important to keep trying to reach out to students even when it seems to not work.

The other half of participants read about how it is difficult to build good relationships with students and that it can potentially backfire. These participants read the following information:

Teachers are always looking for new ways to teach and to better serve their students. One strategy often does not seem to work. While being friends with students sounds good, it can be ineffective and can even make the situation worse. Our own research team has been studying this strategy for combatting misbehavior.

Through interviews and focus groups, teachers have told us that this strategy does not work. Many times, teachers find that it is impossible to get students to listen and understand teachers’ needs, no matter what a teacher does or says. They report that this strategy ultimately wastes time and resources that are better spent on students who behave. They also said that it can backfire. Students’ misbehavior progressively got worse the more teachers tried to reach out to them or level with them. The more teachers tried to show that they cared, the less students respected them.

Participants were then asked to describe a situation when a teacher reached out to a misbehaving student, and it did not work. They were also asked to explain why it might not be possible to get students to cooperate.

#### Measures

All questions were asked on a scale of 1 (not at all) to 5 (extremely). Following each misbehavior, teachers were asked the following questions: (i) How severe was DeShawn’s behavior? (ii) To what extent is DeShawn hindering you from maintaining order in the class? (iii) How irritating is DeShawn? (iv) How severely should DeShawn be disciplined?

Similar to experiment 1, the responses to the first three questions were aggregated into a measure called feeling troubled. After the two misbehaviors, teachers were also asked the following questions: (i) How likely is it that you would say that DeShawn is a troublemaker? (ii) To what extent do you think DeShawn’s behavior is indicative of a pattern? (iii) How likely is it that you will be able to build a strong relationship with DeShawn? (iv) How likely is it that you will refer DeShawn to the principal’s office for disciplinary action in the future? (v) To what extent do you feel personally responsible for DeShawn behaving better in the future?

#### Results

As predicted in preregistration (https://aspredicted.org/blind.php?x=tn885s), independent-samples *t* tests revealed that participants under the treatment condition, as compared to the active control condition, felt less troubled by the Black student’s misbehavior [*t*(254.31) = −3.89, *P* = 0.001, *d* = −0.49, 95% CI (−0.67 to −0.22)], wanted less severe discipline for the student [*t*(254.19) = −4.13, *P* < 0.001, *d* = −0.52, 95% CI (−0.79 to −0.28)], were less likely to label the student as a troublemaker [*t*(252.96) = −1.91, *P* = 0.06, *d* = −0.24, 95% CI (−0.51 to 0.007)], were less likely to think that the misbehavior was indicative of a pattern [*t*(252.57) = −2.69, *P* = 0.008, *d* = −0.34, 95% CI (−0.53 to −0.08)], were more likely to feel able to build a strong relationship with the student [*t*(259.83) = 3.42, *P* < 0.001, *d* = 0.43, 95% CI (0.18 to 0.67)], and were less likely to expect the student to get suspended in the future [*t*(249.72) = −3.01, *P* = 0.003, *d* = −0.38, 95% CI (−0.72 to −0.15)]. See Fig. 2 for graphs of effects on primary dependent variables. See table S2 for means and SDs.

Might these effects be replicated with actual teachers? In addition, might the effects avoid harm to teachers’ responses to White students’ misbehavior?

### Experiment 2

#### Participant information

Teachers were recruited via email from a large school district (serves +100,000 students) in a southern U.S. state. They were given $10 Amazon.com gift cards to compensate for their participation in the study. Because this was a teacher sample, no exclusions were made on the basis of feedback/probes. We made exclusions based on engagement with the materials (i.e., leaving prompts blank) and went from a sample size of 259 to 243 (166 females and 77 males; *M*_age_ = 40.62 and SD = 11.16). The racial breakdown of our sample was as follows: 210 White, 12 Black, 11 Latinx, 6 Asian, and 4 declined to answer. Our sample had a *M*_age_ = 40.63 and SD = 11.16 and average years of teaching experience of *M* = 13.25 and SD = 8.87. Similar to experiment 1 and the pilot study, preregistered hypotheses only delineated predicted relationships after the second misbehavior for the same reasons noted in the prior studies. Our sample from experiment 2 was similar to the K-12 teacher workforce, according to the most recent statistics published by the Department of Education’s Schools and Staff Survey (*28*) (see Table 3).

**Table 3:** Sample comparison between experiment 2 sample and actual K-12 workforce in the United States.

**Characteristic**	**Experiment 2** **sample**	**K-12 teacher** **workforce**
Mean age	41	42
Mean years of experience	13	14
Percent female	68%	77%
Percent White	88%	80%
Percent Hispanic	5%	9%
Percent Black	5%	7%
Percent Asian	3%	2%

#### Procedure

The procedure for experiment 2 was identical to that of the pilot study with the addition of a manipulation of the target student’s race. Teachers were randomly assigned to one of four conditions in the fashion of a 2 (all three treatments versus all three controls) × 2 (student race: Black versus White) between-subjects factorial design. For the student race condition, teachers either read about misbehavior by a Black student (named DeShawn) or a White student (named Greg). These are stereotypical names from previous research that used this paradigm to investigate differences in discipline decisions based on a student’s race (*5*).

#### Measures

All questions were the same as those used in the pilot study, including the rating scale used.

### Statistical analysis

Mixed linear regressions or independent-samples *t* tests were used with R computing software for all analyses reported here. These are standard methods to analyze data with relatively simple 2 × 2 factorial designs with human individuals in social sciences.

## Supplementary Material

aba9479_SM.pdf

## SUPPLEMENTARY MATERIALS

Supplementary material for this article is available at http://advances.sciencemag.org/cgi/content/full/6/42/eaba9479/DC1
