# Supplementary material for: When policy and psychology meet: Mitigating the consequences of bias in schools
Source: Sci Adv. 2020 Oct 16;6(42):eaba9479. doi: 10.1126/sciadv.aba9479 (PMC7567593; doi:10.1126/sciadv.aba9479)
Supplement: aba9479_SM.pdf [file aba9479_SM.pdf]

Science Advances
AAAS

advances.sciencemag.org/cgi/content/full/6/42/eaba9479/DC1

# Supplementary Materials for

## When policy and psychology meet: Mitigating the consequences of bias in schools

Jason A. Okonofua*, Amanda D. Perez, Sean Darling-Hammond

*Corresponding author. Email: okonofua@berkeley.edu

Published 16 October 2020, *Sci. Adv.* **6**, eaba9479 (2020)
DOI: 10.1126/sciadv.aba9479

**This PDF file includes:**

Tables S1 to S3

**Supplemental Materials**

**Data Availability Statement**

All datasets are available at Dryad. Study 1 data: https://doi.org/10.6078/D1VT4T ; Study 2a data: https://doi.org/10.6078/D1R39J; Study 2b data: https://doi.org/10.6078/D1MD7N.

**Code Availability Statement**

All coding scripts are available at Dryad. Study 1 script: https://doi.org/10.6078/D1VT4T ; Study 2a script: https://doi.org/10.6078/D1R39J; Study 2b script: https://doi.org/10.6078/D1MD7N.

**Tables and Figures**

Table S1. Experiment 1 means and standard deviations of the dependent measures broken down by experimental condition. Scales for all outcomes range 1 = Not at all – 5 = Extremely.

| | Feeling Troubled | Student is a Troublemaker | Student is a Danger | Discipline-severity | Future Strong Relationship Likelihood | Behavior is a Pattern |
|---|---|---|---|---|---|---|
| Perspective and Growth | M = 2.28 SD = 0.62 | M = 1.80 SD = 0.78 | M = 1.20 SD = 0.40 | M = 1.83 SD = 0.76 | M = 3.63 SD = 0.78 | M = 3.17 SD = 0.96 |
| Perspective and no Growth | M = 2.69 SD = 0.90 | M = 2.35 SD = 1.07 | M = 1.58 SD = 0.78 | M = 2.24 SD = 0.95 | M = 3.31 SD = 1.01 | M = 3.39 SD = 1.06 |
| Growth and no Perspective | M = 2.51 SD = 0.78 | M = 2.29 SD = 1.06 | M = 1.55 SD = 0.68 | M = 2.09 SD = 0.92 | M = 3.62 SD = 1.04 | M = 3.32 SD = 1.00 |
| No Growth and no Perspective | M = 2.47 SD = 0.81 | M = 2.22 SD = 1.07 | M = 1.55 SD = 0.73 | M = 2.20 SD = 0.92 | M = 3.32 SD = 1.04 | M = 3.23 SD = 1.14 |

Table S2. Pilot study means and standard deviations of the dependent measures broken down by experimental condition. Scales for all outcomes range 1 = Not at all – 5 = Extremely.

| | Feeling Troubled | Discipline-severity | Student is a Troublemaker | Behavior is a Pattern | Future Strong Relationship Likelihood | Future Discipline Likelihood | Feeling Personally Responsible |
|---|---|---|---|---|---|---|---|
| Teacher Growth | M = 3.43 SD = 0.96 | M = 3.00 SD = 1.02 | M = 3.05 SD = 1.07 | M = 3.63 SD = 0.94 | M = 2.82 SD = 0.95 | M = 3.15 SD = 1.09 | M = 2.87 SD = 1.16 |
| Active Teacher Control | M = 3.87 SD = 0.88 | M = 3.53 SD = 1.04 | M = 3.30 SD = 1.02 | M = 3.94 SD = 0.87 | M = 2.40 SD = 1.04 | M = 3.59 SD = 1.22 | M = 2.55 SD = 1.17 |

Table S3. Experiment 2 means and standard deviations of the dependent measures broken down by experimental condition. Scales for all outcomes range 1 = Not at all – 5 = Extremely.

| | Feeling Troubled | Discipline-severity | Student is a Troublemaker | Behavior is a Pattern | Future Strong Relationship Likelihood | Future Discipline Likelihood | Feeling Personally Responsible |
|---|---|---|---|---|---|---|---|
| Teacher Growth with Black Student | M = 2.78 SD = 0.93 | M = 2.42 SD = 0.95 | M = 2.07 SD = 0.93 | M = 3.02 SD = 0.98 | M = 3.39 SD = 0.88 | M = 2.21 SD = 0.94 | M = 3.10 SD = 1.00 |
| Active Teacher Control with Black Student | M = 3.01 SD = 0.89 | M = 2.71 SD = 0.85 | M = 2.52 SD = 1.00 | M = 3.36 SD = 1.01 | M = 2.85 SD = 0.86 | M = 2.75 SD = 1.14 | M = 2.89 SD = 1.05 |
| Teacher Growth with White Student | M = 2.62 SD = 0.99 | M = 2.20 SD = 0.93 | M = 2.10 SD = 1.01 | M = 3.15 SD = 0.93 | M = 3.05 SD = 0.87 | M = 2.29 SD = 1.05 | M = 2.97 SD = 0.91 |
| Active Teacher Control with White Student | M = 2.64 SD = 0.96 | M = 2.35 SD = 1.02 | M = 2.18 SD = 0.97 | M = 3.05 SD = 1.14 | M = 3.38 SD = 0.91 | M = 2.47 SD = 1.16 | M = 2.73 SD = 1.12 |

**Study 1**

*Exploratory analyses*. In Experiment 1, teachers were asked how dangerous they thought the student would be to other students at the school. A linear regression revealed a significant interaction of the interventions such that – when combined (student-perspective and student-growth) – they led teachers, after the second misbehavior, to be less likely to view the student as a danger (b = -0.10, se = 0.04, $t(3, 242) = -2.28$, $p = .02$), as compared to any other condition.
